# Analysis of influence of deformation modes of retaining structures on deformation of a side shield tunnel

**DOI:** 10.1038/s41598-022-24534-6

**Published:** 2022-11-20

**Authors:** Gang Wei, Binglai Guo, Zhe Wang, Hongguo Diao, Xinquan Wang

**Affiliations:** 1 Department of Civil Engineering, Zhejiang University City College, Hangzhou, 310015 China; 2 Key Laboratory of Safe Construction and Intelligent Maintenance for Urban Shield Tunnels of Zhejiang Province, Hangzhou, 310015 China; 3 Zhejiang Engineering Research Center of Intelligent Urban Infrastructure, Hangzhou, 310015 China; 4 Institute of Geotechnical Engineering, Zhejiang University of Technology, Hangzhou, 310014 China

**Keywords:** Civil engineering, Mathematics and computing

## Abstract

In the process of foundation pit excavation, different retaining structures and support stiffness may lead to different deformation modes of retaining structures. The soil displacement cause by the deformation of the retaining structure is calculated by the virtual image technique. Meanwhile, the collaborative deformation model for rotation and dislocation is introduced to analyze the tunnel longitudinal deformation caused by different deformation modes as well as the tunnel maximum displacement equivalent field, and two case verifications are carried out. The study shows that there is a large difference in the size and distribution law of the soil displacement field caused by different enclosure structure deformation modes. The horizontal displacement of soil caused by the cantilever type always shows a "cantilever type" curve with increasing horizontal distance from the enclosure structure, while the composite type, inner convex type and kicker type develop from the "bow" to the "cantilever type" curve. The vertical displacement field of the soil is in the shape of a "spoon" and the soil exhibits a certain bulge deformation below the critical depth. The critical depths of the composite type and inner convex type are similar, while the kick type is the largest and the cantilever type is the smallest. The influence area of the maximum horizontal displacement of the tunnel outside the pit of the inner convex type and the composite type is basically the same. The influence area of the cantilever mode is the smallest, while the influence depth of the kick-in mode is higher than that of the other three deformation modes.

## Introduction

With the continuing development of rail transit, excavation projects of foundations adjacent to shield tunnels are becoming more common. For example, the minimum horizontal distance between the metro and the foundation pit was only 18.1 m^1^. The closest distance was approximately 33 m between a construction foundation pit and Nanning Metro Line 2^2^. The unloading effect on the sidewall due to the excavation of the foundation pit leads to the deformation of the enclosure structure, which generates additional loads on the adjacent tunnel. The excessive loads will endanger the safety of the tunnel. An engineering case in which the tunnel section of the Panchiao line was damaged due to adjacent foundation pit excavation during the construction of the Taipei MRT system was reported^3^. As a result, it is important to predict the deformation of adjacent shield tunnels due to foundation pit excavation.

This kind of engineering problem has received attention at home and abroad. At present, the main research methods are theoretical calculation^4–6^, numerical simulation^7^, centrifuge testing^8^ and measured data analysis^9,10^. In theoretical calculation, the two-stage analysis method is the most widely used method: first, calculating the soil unloading stress or displacement caused by the foundation excavation, then, computing the deformation of the tunnel subjected additional stress or displacement. For the unloading stress on the sidewall of the pit caused by the excavation of the pit, most of the current studies consider the active earth pressure outside the pit as the sidewall unloading amount^11,12^, the support function of the enclosure cannot be considered. Some literatures^13,14^ use the unloading loss rate *β* to consider the partial release of unloading stresses in the sidewalls. However, during the excavation of the foundation pit, the enclosure is bound to deform to varying degrees, thus affecting the amount of unloaded load on the enclosure. Zhang et al.^15^ derived the formula for calculating the unloading capacity considering the displacement under the convexity deformation pattern. It has been shown that there are differences in unloading on the sidewalls for different modes of envelope deformation, thus having different effects on the shield tunnel outside the pit^16^. However, studies on sidewall unloading under other deformation modes of the envelope have not been reported.

In the presented analytical methods, the shield tunnels are mainly considered as Euler Bernoulli beams as a way to simulate the bending deformation of the tunnel^4,5^. However, the Euler–Bernoulli beam cannot take into account the shear effect between the rings, and thus it leads to a significant overestimation of the shear effect. The Timoshenko beam, which takes into account both bending and shear effects, are also commonly used to simulate shield tunnels^6,13^. However, the Timoshenko beam is still unable to simulate the assembling characteristics of segment rings. To fully understand the tunnel deformation mechanism, a few studies^15,17^ built a collaborative deformation mode with rotation and dislocation to simulate the shield tunnel.

This paper summarizes the formulas for calculating the deformation curve of pit sidewalls under four typical enclosure deformation modes. Meanwhile, the soil displacement fields caused by different enclosure deformation modes are calculated based on the virtual image technique, and the horizontal action range and influence depth of different enclosure deformation modes are compared and analyzed. In addition, this paper further introduces a rotating staggered platform collaborative deformation tunnel model that can fully take into account the tube assembly characteristics and investigates the difference in longitudinal deformation of the side-by-side shield tunnel under different pit envelope deformation modes.

## Deformation pattern and prediction curve of foundation pit enclosure structure

**Figure 1:**
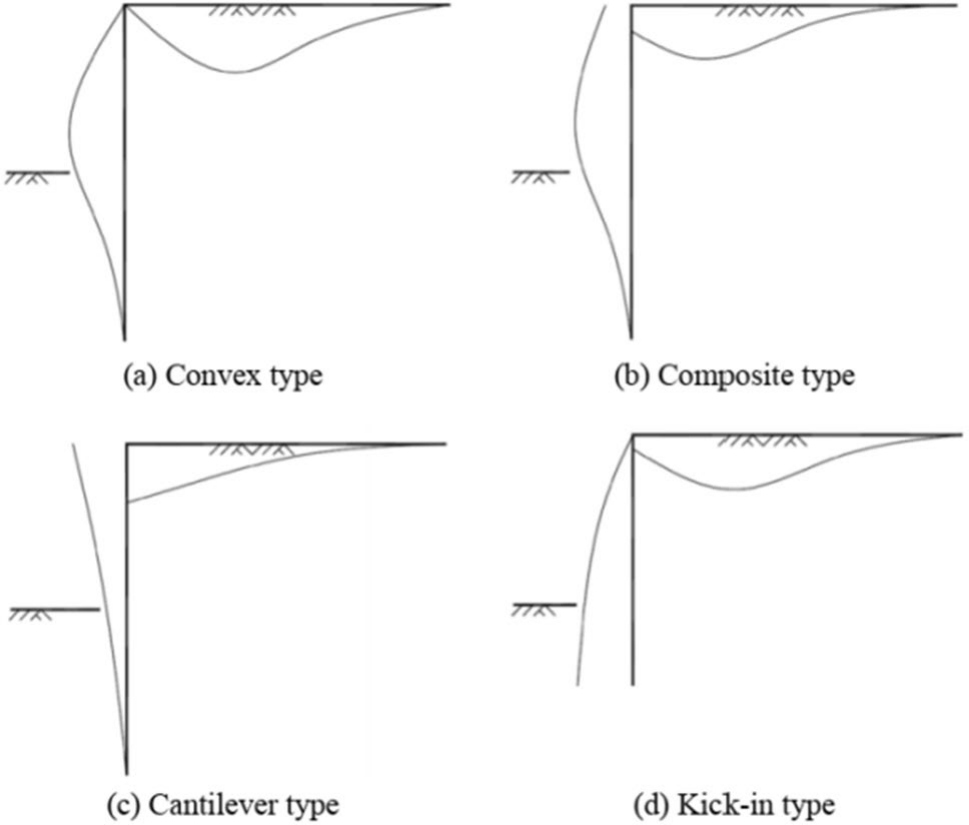
Typical profiles of deflection of retaining structures^18^.

The deformation mode of a foundation pit enclosure structure is influenced by the form of foundation support and construction methods. There are four main deformation modes: cantilever, kick-in, convex and composite^18^. The four deformation modes are shown schematically in Fig. 1.

### Cantilever-type deformation mode of enclosure structure

For flexible enclosure structures, when no support is set or excavation is shallow and no support has yet been set, there is a cantilevered distribution with maximum top displacement^19^.

**Figure 2:**
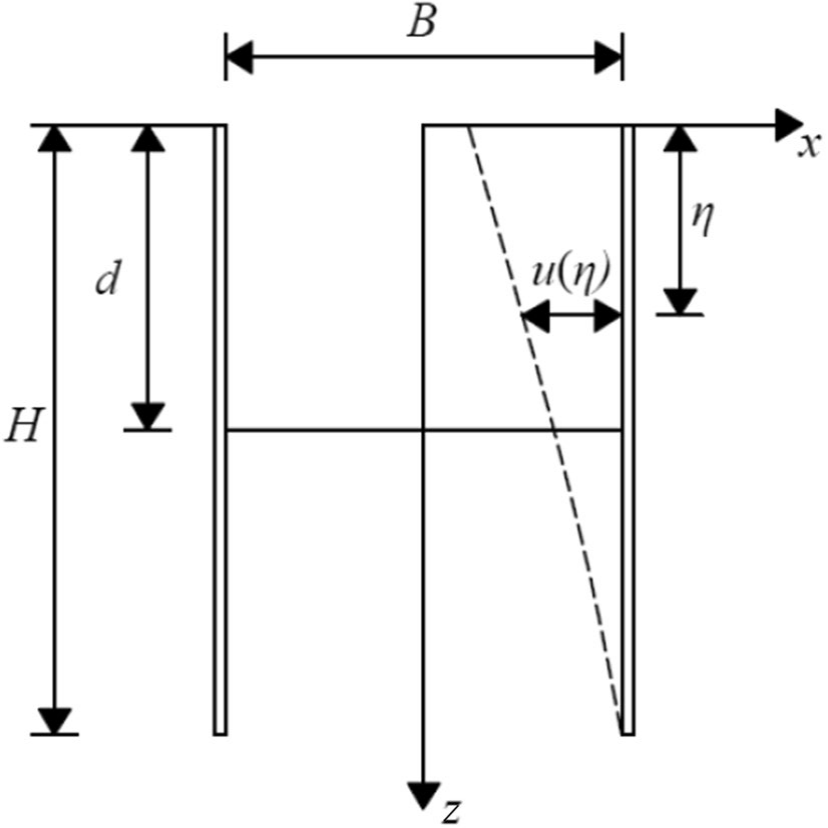
Calculation model of cantilever deformation.

As indicated in Fig. 2, the width of the pit is *B*, the excavation depth is *d*, and the depth of the enclosure structure is *H*. The enclosure structure underwent cantilever deformation. The deformation of enclosure structure at depth *η* is *u*(*η*). Gu et al.^20^ simplified the deformation of the row pile support to the cantilever deformation mode and provided the deformation curve of the row pile. Several scholars^21–23^ used the same hypothetical curve as the cantilever deformation of the enclosure structure.

1 $$ u(z) = \frac{{\delta_{\max } }}{2}\sin \frac{{\uppi }}{L}y\left( {1 + \cos \frac{{\uppi }}{H}z} \right) $$

where *u* denotes the displacement of the sidewall to the pit at depth *z* from corner *y* of the pit, *H* indicates the length of the enclosure structure, *L* corresponds to the length of the pit, and $\delta_{\max }$ represents the maximum displacement of the sidewall.

When *y* = *L*/2, substituting into Eq. (1), we obtain the deformation curve of the sidewall in the middle of the pit:

2 $$ u(z) = \frac{{\delta_{\max } }}{2}\left( {1 + \cos \frac{{\uppi }}{H}z} \right) $$

### Kick-in type deformation mode of enclosure structure

For the enclosure structure with the bottom of the wall located in soft soil, if the insertion depth is shallow, then the bottom of the wall will have a large displacement, which manifests as a kick-in deformation pattern^19^.

**Figure 3:**
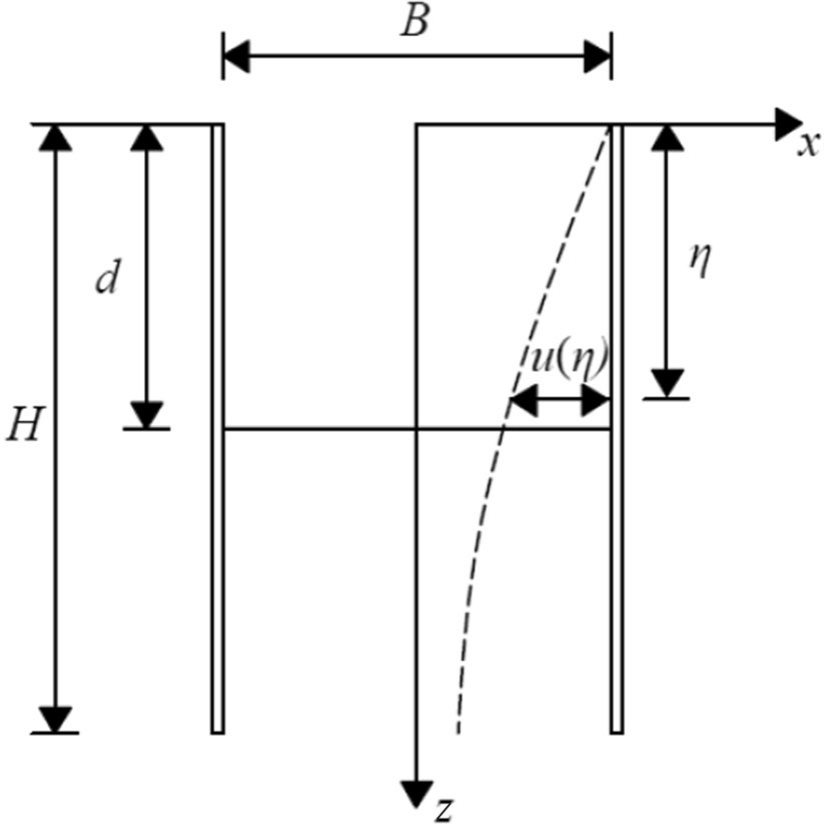
Calculation model of kick-in deformation.

As depicted in Fig. 3, the enclosure structure undergoes a kick-in-type deformation mode. Ju et al.^24^ set the constraint of a fixed top and flat bottom to simulate the kick-in deformation mode and fitted the enclosure deformation curve with polynomials to obtain the formula for calculating the horizontal displacement of the enclosure structure under the kick-in deformation mode as follows:

3 $$ u(z){ = }\alpha \left( \frac{z}{H} \right)\left( {\frac{3z}{{2H}} - \frac{{z^{2} }}{{H^{2} }}} \right) $$

where *α* denotes the coefficient to be determined, and the specific value was obtained from the literature^25^.

Because the bottom deformation of the envelope in the skirting-type deformation mode reaches the maximum value, that is, $u(H) = \delta_{\max }$, substituting into Eq. (3) yields $\alpha = 2\delta_{\max }$. Equation (3) can be rewritten as follows:

4 $$ u(z){ = }2\delta_{\max } \left( \frac{z}{H} \right)\left( {\frac{3z}{{2H}} - \frac{{z^{2} }}{{H^{2} }}} \right) $$

### Convex type deformation mode of envelope structure

**Figure 4:**
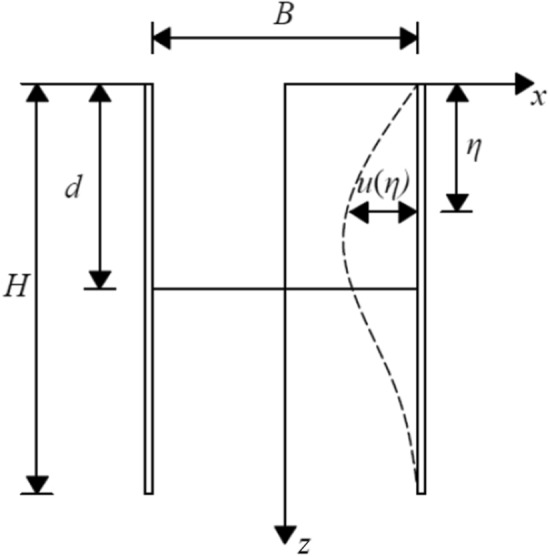
Calculation model of convex deformation.

As indicated in Fig. 4, Zhang et al.^15^ fitted the enclosure deformation increment with a segmental cosine function and obtained the deformation curve of the convex type deformation mode.

5 $$ \left. \begin{gathered} \delta_{i} \left( {z,d_{i} } \right){ = }\frac{{\delta_{\max i} }}{2}\left[ {1 - \cos \left( {\frac{{{\uppi }z}}{{d_{i} }}} \right)} \right]\mathop {}\nolimits^{{}} \mathop {}\nolimits^{{}} \mathop {}\nolimits^{{}} \mathop {}\nolimits^{{}} \mathop {}\nolimits^{{}} \mathop {}\nolimits^{{}} \mathop {}\nolimits^{{}} \mathop {}\nolimits^{{}} \left( {0 \le z \le d_{i} } \right) \hfill \\ \delta_{i} \left( {z,d_{i} } \right){ = }\frac{{\delta_{\max i} }}{2}\left\{ {1 - \cos \left\{ {\frac{{{\uppi }\left[ {z + (H - 2d_{i} )} \right]}}{{H - d_{i} }}} \right\}} \right\}\mathop {}\nolimits^{{}} \mathop {}\nolimits^{{}} \left( {d_{i} \le z \le H} \right) \hfill \\ \end{gathered} \right\} $$

where *δ*_*i*_(*z*, *d*_*i*_) indicates the incremental deformation of the pit sidewall enclosure at depth *z* caused by the excavation of the *i*th layer, and di denotes the depth of the excavation surface after the excavation of the *i*th layer. Moreover, *δ*_max *i*_ denotes the maximum incremental deformation of the pit sidewall enclosure caused by the excavation of the *i*th layer.

Zhang et al.^15^ used the ratio of the accumulated maximum deformation of the enclosure to the excavation depth *δ*_max_/*d* as the control parameter of the enclosure deformation, and they assumed that the accumulated deformation of each layer of excavation satisfies the control value. Accordingly, the maximum deformation increment of the excavation of the *i*th layer can be expressed as follows:

6 $$ \left. {\begin{array}{*{20}l} {\delta_{\max i} { = }\frac{{\delta_{\max } }}{d} \cdot d_{i} } \hfill & {\left( {i{ = }1} \right)} \hfill \\ {\delta_{\max i} { = }\frac{{\delta_{\max } }}{d} \cdot d_{i} - \sum\limits_{j = 1}^{i - 1} {\delta_{j} \left( {d_{i} } \right)} } \hfill & {\left( {i \ge 2} \right)} \hfill \\ \end{array} } \right\} $$

When the foundation pit is excavated in *n* layers and the depth of the excavated surface reaches *d*_*i*_, the cumulative deformation distribution of the enclosure structure can be expressed as follows:

7 $$ u\left( z \right) = \sum\limits_{i = 1}^{n} {\delta_{i} \left( {z,d_{i} } \right)} $$

where the excavation depth of the foundation pit represents the sum of the thicknesses of each previous excavation layer, $d = d_{{1}} { + }\sum\limits_{i = 2}^{n} {\left( {d_{i} { - }d_{i - 1} } \right)}$.

### Composite-type deformation mode of envelope structure

**Figure 5:**
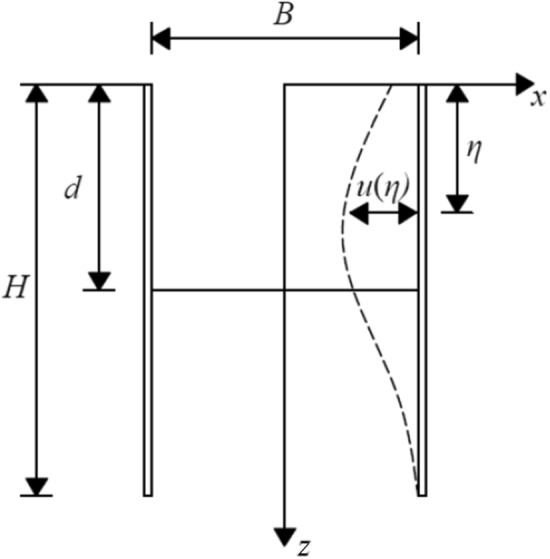
Calculation model of composite deformation.

Figure 5 presents a schematic of the composite deformation of the enclosure structure. Based on the actual measurement data of typical deep-foundation pit projects, Cai et al.^25^ provided a formula for the lateral deformation curve of the enclosure wall on the symmetrical surface of the pit. Zhang et al.^26^ and Zhu et al.^27^ also used the formula to predict the composite deformation of the enclosure structure, which is as follows:

8 $$ u(z) = \delta_{\max } \cdot e^{{ - 1.5\left( {\frac{z - H}{H}} \right)^{2} }} $$

## Analysis of soil displacement on outside of foundation pit

### Soil displacement calculation formula

In 1987, Sagasteta^28^ proposed the virtual image technique to derive a formula to mathematically describe the distribution law of the surrounding soil displacement field owing to stratigraphic losses at any point in the elastic half-space. In this study, that method was used to calculate the soil displacement caused by the deformation of the pit sidewalls. The displacement mechanism of the soil loss involves certain simplifications with the following assumptions: (a) The soil is incompressible, and the soil displacement field is only due to soil loss. (b) The calculation process does not consider pore water pressure, soil consolidation, etc. (c) The same size of deformation occurs in each section of the envelope structure.

**Figure 6:**
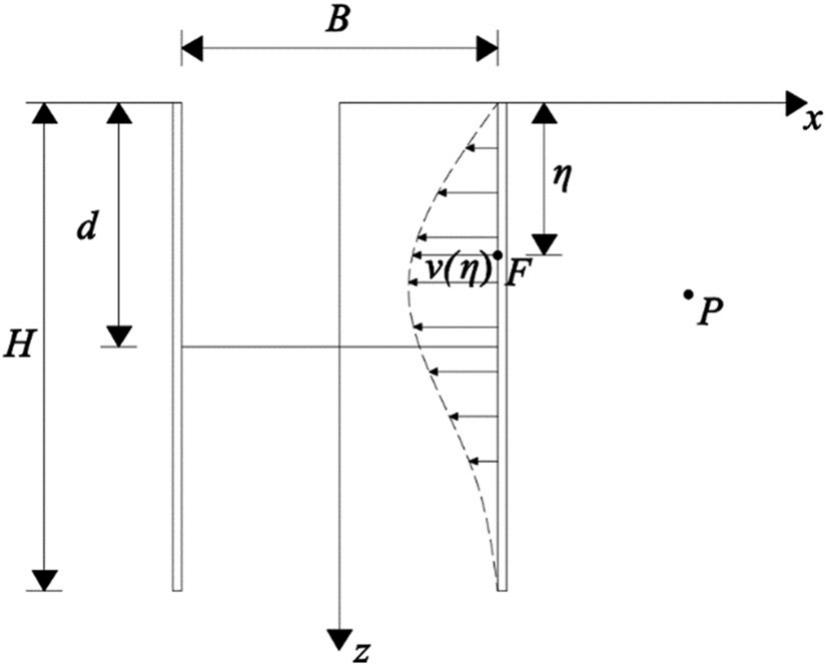
Calculation model of virtual image technique.

Taking the convex deformation model as an example, Fig. 6 shows a calculation model of the virtual image technique. The displacement along the *x*-axis component of the gap of radius *a* at the point $F(B/2,\eta )$ generated at point $P(x_{1} ,z_{1} )$ on the axis of the tunnel is as follows:

9 $$ S_{x1} = - \frac{a2}{2}\frac{{x_{1} - B/2}}{{r_{1} 2}} $$

where $r_{1} = \sqrt {(B/2 - x_{1} )^{2} + (\eta - z_{1} )^{2} }$ indicates the distance between the point *F* and the point *P*.

Equation (9) represents the displacement expression in an infinite space, and the construction site is a semi-infinite space. Therefore, it is necessary to transform the solution problem of an infinite body into that of a semi-infinite space. Accordingly, $F(B/2,\eta )$ is mirrored as $F^{^{\prime}} (B/2, - \eta )$, and a volume expansion of equal size occurs at the point. The displacement generated by $F^{^{\prime}} (B/2, - \eta )$ at point $P(x_{1} ,z_{1} )$ along the *x*-axis component is as follows:

10 $$ S_{x2} = \frac{a2}{2}\frac{{x_{1} - B/2}}{{r_{2} 2}} $$

where $r_{2} = \sqrt {(B/2 - x_{1} )^{2} + (\eta { + }z_{1} )^{2} }$ indicates the distance between the point *F′* and the point *P*.

During the establishment of Eq. (9), a shear strain is generated at the ground level.

11 $$ \gamma { = } - 4a^{2} \frac{{\eta (x_{1} - B/2)}}{{\left[ {(x_{1} - B/2)^{2} + \eta^{2} } \right]^{2} }} $$

The additional shear stress generated at the ground surface is expressed as follows:

12 $$ \tau = G\gamma = - 4Ga^{2} \frac{{\eta (x_{1} - B/2)}}{{\left[ {(x_{1} - B/2)^{2} + \eta^{2} } \right]^{2} }} $$

where *G* denotes the soil shear modulus.

When additional shear stress is applied to the surface with the opposite sign, the displacement component along the *x*-axis generated by the shear stress at the point *P* is expressed as follows:

13 $$ S_{x3} = - \frac{{a^{2} (x_{1} - B/2)}}{{r_{2}^{2} }}\left[ {1 - 2\frac{{z_{1} (z_{1} + \eta )}}{{r_{2}^{2} }}} \right] $$

In summary, the component of the soil displacement along the *x*-axis generated by a void of radius *a* at the point *F* in a semi-infinite space at the point *P* is as follows:

14 $$ S_{x} (z_{1} ,\eta ) = S_{x1} + S_{x2} + S_{x3} $$

Since the outside of the enclosure should be 1/2 half-space, based on the symmetry, the problem can be transformed into a solution of deformation at any point under the stratigraphic loss of 2*v* generated in the elastic half-space space, as shown in Fig. 6. Accordingly, the horizontal displacement of the enclosure is divided equally into *n* differential segments, each of which is approximated as a rectangle. The area corresponding to each differential segment is equated to a circle based on the area equivalence principle, and the equivalence radius was $a = \sqrt {2vd\eta /\pi }$. By integrating the excavation depth, we can obtain the horizontal displacement of the soil produced by the deformation of the section of the enclosure structure shown in Fig. 6 for $P(x_{1} ,z_{1} )$:

15 $$ S_{{\text{x}}}^{^{\prime}} (x_{1} ,z_{1} ) = \int_{0}^{H} {\frac{2v}{\pi }} \left\{ { - \frac{1}{2}\left( {\frac{{x_{1} - B/2}}{{r_{1}^{2} }} - \frac{{x_{1} - B/2}}{{r_{2}^{2} }}} \right) - \frac{{x_{1} - B/2}}{{r_{2}^{2} }}\left[ {1 - 2\frac{{z_{1} (z_{1} + \eta )}}{{r_{2}^{2} }}} \right]} \right\}d\eta $$

Similarly, the vertical displacement of the soil caused by the section deformation of the envelope structure is

16 $$ S_{z}^{^{\prime}} (x_{1} ,z_{1} ) = \int_{0}^{H} {\frac{2v}{\pi }} \left\{ { - \frac{1}{2}\left( {\frac{{z_{1} - \eta }}{{r_{1}^{2} }} - \frac{{z_{1} + \eta }}{{r_{2}^{2} }}} \right)} \right\}d\eta $$

## Analysis of soil displacement characteristics in different deformation modes of foundation pit enclosures

During the excavation of the foundation pit, the current relevant specifications^29,30^ all control the maximum deformation of the enclosure structure. However, even if the maximum deformation of the enclosure structure is the same, the displacement field of the soil outside the pit will be different due to the different deformation modes of the enclosure structure. Therefore, it is of great significance for practical engineering to analyze the difference in soil displacement caused by different enclosure structures under the same maximum deformation of the foundation pit enclosure.

### Case analysis

Take the deep foundation pit project next to the shield tunnel of Metro Line 2 that has been put into operation at the intersection of Shixin Road and Jincheng Road in Xiaoshan District, Hangzhou City as a case. The excavation size of the foundation pit on the side of the tunnel is *L* = 68 m, *B* = 72 m, excavation depth *d* = 15.8 m, underground diaphragm wall is 37.2 m below the ground, and the minimum distance between the sideline of the foundation pit enclosure and the tunnel axis is *s* = 12.6 m^31^. Statistics of the measured data show^32–34^ that the excavation depth of the foundation pit is closely related to the accumulated maximum deformation of the foundation pit enclosure. The *δ*_max_/*d* of the foundation pit enclosure in Hangzhou varies from 0.09 to 0.61%^32^. The deformation of foundation pits in Shanghai calculated by Xu et al.^33^ is very close to that collected by Qu et al.^34^ in Taipei, China, with *δ*_max_/*d* ranging between 0.1 and 1.0%, with an average value of 0.42%.

**Figure 7:**
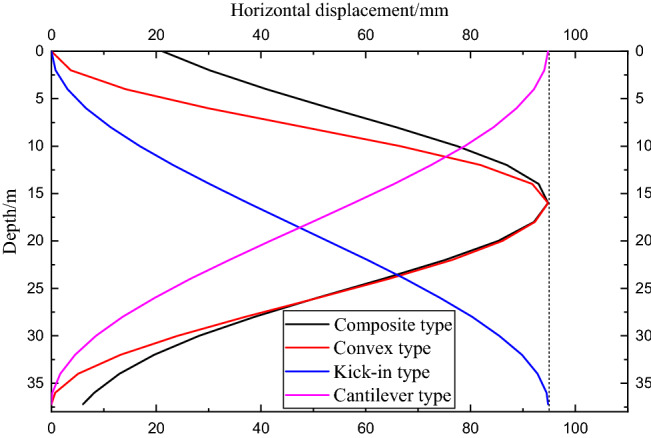
Deformation curves of sidewall under different deformation modes.

The four envelope deformation modes *δ*_max_/*d* of this project case are taken as 0.6%. Under the condition of the same cumulative maximum deformation, the deformation curves of the sidewall under the four deformation modes of the foundation pit enclosure are shown in Fig. 7.

**Figure 8:**
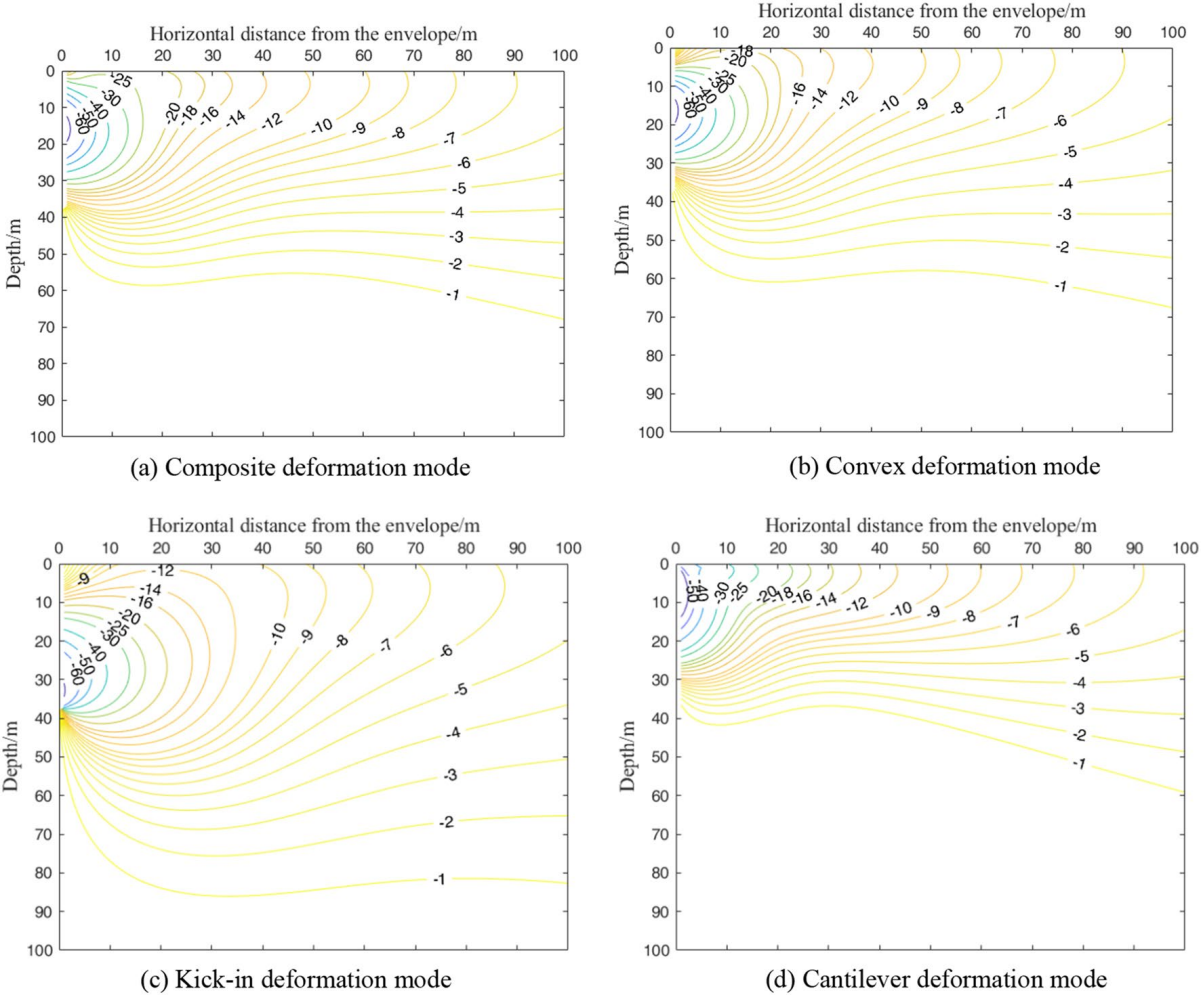
Horizontal displacement field of soil caused by different deformation modes of retaining structure.

Figure 8 shows the horizontal displacement field of the soil generated by the four deformation modes of the foundation pit envelope. In Fig. 8, the corresponding soil deformation is marked by color, the negative sign indicates that the soil moves into the foundation pit, and the unit is mm.

As shown in Fig. 8a and b, the soil horizontal displacement field generated by the convex deformation mode and the composite deformation mode is similar, and the soil horizontal displacement decreases with increasing distance from the enclosure structure. When the distance to the enclosure structure is relatively short (less than 10 m), the horizontal displacement curve of the soil exhibits an obvious "bow shape". In addition, the horizontal displacement of the surface increases with increasing distance from the enclosure structure, while the horizontal displacement of the deep layer shows a decreasing law. With a further increase in the horizontal distance from the enclosure structure, the horizontal displacement curve of the soil transitions to the "cantilever type" deformation curve, the horizontal displacement of the surface gradually decreases, and the curve tends to be gentle. The impact gradually diminishes. With the increase in the horizontal distance from the enclosure structure, the horizontal displacement distribution of the soil generated by the composite deformation mode enters the "cantilever type" distribution law earlier than the convex deformation mode, the horizontal influence range is wider, and the influence depth is roughly the same.

Figure 8c shows the horizontal displacement field of the soil caused by the kick-in deformation mode of the enclosure structure. Affected by the deformation of the enclosure structure, the soil close to the enclosure has the same deformation as the enclosure, showing a kick-type deformation. The horizontal displacement of the soil near the bottom of the wall is the largest, while it decreases rapidly below the bottom of the wall. When the horizontal distance from the envelope structure is small, the horizontal displacement of the soil also shows a "bow shape." However, the maximum deformation occurs near the bottom of the enclosure. With an increase in the horizontal distance from the enclosure, the horizontal displacement of the soil gradually develops into a "cantilever type." Compared with the composite and convex deformation modes, the kick-in deformation mode has a deeper influence on the horizontal displacement of the soil.

The horizontal soil displacement field generated by the cantilever deformation mode of the envelope structure is shown in Fig. 8d. Different from other deformation modes, the soil horizontal displacement curve generated by the cantilever deformation mode always presents a "cantilever type"; the horizontal displacement of the surface is the largest, and the horizontal displacement gradually decreases with increasing depth. As the distance from the enclosure structure increases, the soil displacement curve gradually becomes flat.

**Figure 9:**
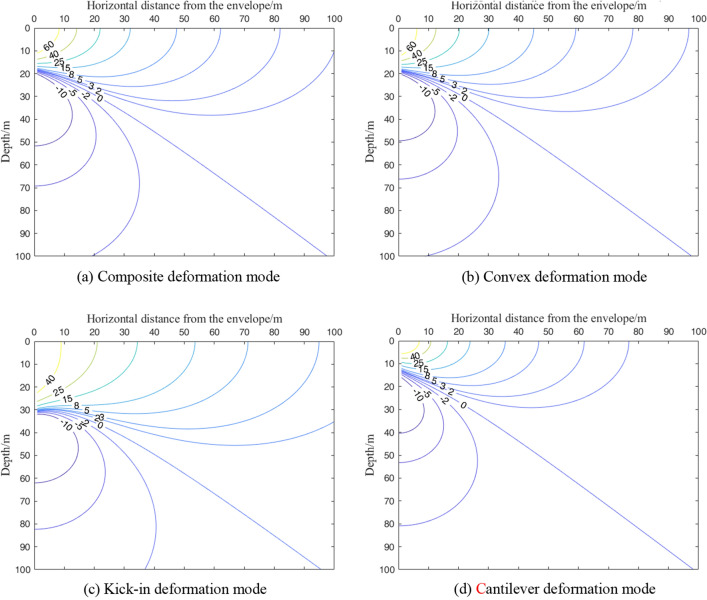
Vertical displacement field of soil caused by different deformation modes of retaining structure.

Figure 9 shows the vertical displacement fields of the soil generated by the four deformation modes of the foundation pit enclosure. In the figure, the corresponding soil deformations are marked by color, the positive sign indicates the settlement of the soil, and the unit is mm.

As shown in Fig. 9a and b, the vertical displacement field of the soil generated by the composite deformation mode is similar to that of the convex deformation mode, and the influence range of the convex deformation mode is slightly smaller than that of the composite mode. Taking the surface soil settlement value of 2 mm as an example, the position where the 2-mm surface settlement occurs is approximately 82 m away from the enclosure structure in the composite mode, and it is approximately 78 m in the convex mode. The vertical displacement decreases gradually with increasing distance from the envelope.

The soil settlement outside the pit presents a "spoon"-shaped distribution. Figure 9c shows the vertical displacement field of the soil caused by the kick-in deformation mode of the enclosure structure. Since the bottom of the enclosure structure in the kick-in deformation mode reaches the maximum deformation value, the influence range of the soil settlement outside the pit caused by this mode is the largest.

The horizontal displacement field of soil caused by the cantilever deformation mode of the envelope structure is shown in Fig. 9d. In the kick-in mode, the position where the 2-mm surface settlement occurs is approximately 61 m away from the enclosure structure, and the influence range of the soil settlement outside the pit caused by the cantilever deformation mode is the smallest. This is related to the maximum deformation of the enclosure structure at the top of the cantilever deformation mode.

Under the four deformation modes of the foundation pit enclosure, the soil will have a certain degree of uplift below a certain depth. The critical depth is related to the deformation mode of the envelope structure. The critical depth of the composite type is similar to that of the convex mode, the kick-in mode is the largest, and the cantilever mode is the smallest.

## Analysis of longitudinal displacement of shield tunnel outside foundation pit

### Calculation of horizontal displacement of shield tunnel outside foundation pit

#### Collaborative deformation model of shield tunnel with rotation and dislocation

This paper cites a cooperative deformation model that can comprehensively consider the two deformation effects of segment ring rotation and dislocation of shield tunnels^15^.

**Figure 10:**
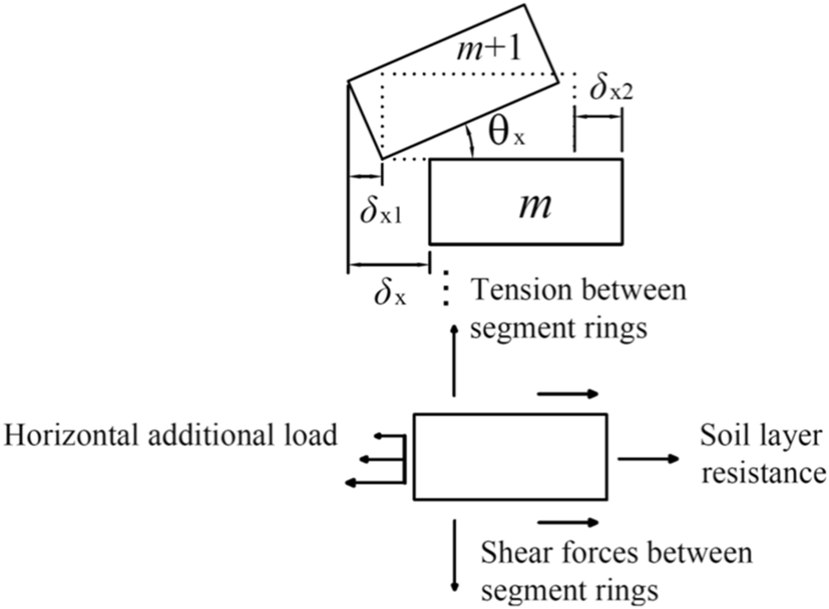
Calculation model for collaborative deformation of rotation and dislocation between shield tunnel segment rings^15^.

As shown in Fig. 10, relative rotation and relative dislocation will occur between adjacent segments. The longitudinal deformation of the shield tunnel is considered to be formed by combining shear dislocation and rigid body rotation between adjacent segments.

Assuming that the shield tunnel beside the foundation pit and the surrounding soil meet the deformation coordination conditions, the displacement of the tunnel is equal to the displacement value of the soil at the corresponding position:

17 $$ w{(}l{)} = w_{{\text{t}}} {(}l{)} $$

where *l* is the calculated longitudinal position of the tunnel, *w*(*l*) is the horizontal displacement distribution of the shield tunnel beside the foundation pit, and *w*_t_(*l*) is the horizontal displacement of the soil around the tunnel.

The horizontal displacement between rings can be expressed as

18 $$ \delta_{{\text{x}}} = w\left( {(m + 1)D_{{\text{t}}} } \right) - w\left( {mD_{{\text{t}}} } \right){ = }w_{{\text{t}}} \left( {\left( {m + 1} \right)D_{{\text{t}}} } \right) - w_{{\text{t}}} \left( {mD_{{\text{t}}} } \right) $$

where *m* and *m* + 1 are the serial numbers of the two adjacent segment rings, and *D*_t_ is the ring width of the segment rings.

#### Shield tunnel deformation total potential energy

According to the process analysis of the longitudinal deformation of the shield tunnel, the total potential energy of the shield tunnel deformation can be specifically composed of the following four parts:

(1) Work done by horizontal additional loads caused by soil deformation:
  19 $$ W_{{\text{L}}} = \sum\limits_{m = - N}^{N - 1} {\int_{{mD_{{\text{t}}} }}^{{(m + 1)D_{{\text{t}}} }} {w(l)} } P_{{{\text{ax}}}} (l){\text{d}}l = \int_{{ - ND_{{\text{t}}} }}^{{ND_{{\text{t}}} }} {w(l)} P_{{{\text{ax}}}} (l){\text{d}}l $$
  where $$\left\{ \begin{gathered} P_{{{\text{ax}}}} (l) = kDS_{{\text{x}}}^{^{\prime}} L,\left| l \right| \le L/2 \hfill \\ P_{{{\text{ax}}}} (l) = 0,\begin{array}{*{20}c} {} & {} \\ \end{array} \left| l \right| \ge L/2 \hfill \\ \end{gathered} \right.$$, and 2*N* is the number of segment rings within the calculation range of the shield tunnel. In theory, the larger the value of *N*, the higher the calculation accuracy. However, the corresponding calculation amount will also increase, and the calculation efficiency will be affected.
Overcome the resistance of the formation to do work:
  20 $$ W_{{\text{R}}} = - \sum\limits_{m = - N}^{N - 1} {\int_{{mD_{{\text{t}}} }}^{{(m + 1)D_{{\text{t}}} }} {\frac{1}{2}w(l)} } kDw(l){\text{d}}l{ = } - \int_{{ - ND_{{\text{t}}} }}^{{ND_{{\text{t}}} }} \frac{1}{2} kD\left[ {w(l)} \right]^{2} {\text{d}}l $$
Work against interring shear force:
  21 $$ W_{{\text{S}}} = - \sum\limits_{m = - N}^{N - 1} {\frac{1}{2}Q_{{\text{x}}} \delta_{{{\text{x}}2}} } { = } - \sum\limits_{m = - N}^{N - 1} {\frac{1}{2}k_{{{\text{sl}}}} \left( {1 - j_{{\text{x}}} } \right)^{2} \delta_{{\text{x}}}^{2} } $$
Overcome the tension caused by the rotation angle between the rings to do work:
  22 $$ W_{{\text{T}}} = - \sum\limits_{m = - N}^{N - 1} {\left( {\int_{r = 0}^{r = D} {\frac{1}{2}\frac{{k_{{\text{t}}} }}{D}\theta_{{\text{x}}}^{2} r^{2} {\text{d}}r} } \right)} = - \sum\limits_{m = - N}^{N - 1} {\frac{{k_{{\text{t}}} \theta_{{\text{x}}}^{2} D^{2} }}{6}} $$

Therefore, the following can be obtained:

23 $$ W_{{\text{T}}} = - \sum\limits_{m = - N}^{N - 1} {\frac{{k_{{\text{t}}} j_{{\text{x}}}^{2} D^{2} }}{{6D_{{\text{t}}}^{2} }}} \left[ {w\left( {(m + 1)D_{{\text{t}}} } \right) - w\left( {mD_{{\text{t}}} } \right)} \right]^{2} $$

The total potential energy of deformation of the shield tunnel beside the foundation pit is

24 $$ E_{{\text{p}}} = W_{{\text{L}}} + W_{{\text{R}}} + W_{{\text{S}}} + W_{{\text{T}}} $$

In Eqs. (19)–(22), *k*_sl_ and *k*_t_ are the interring shear stiffness and interring tensile stiffness of the tunnel, respectively^35^. *k* is the subgrade coefficient of the soil, calculated by the Vesic^36^ formula, $k = \frac{{0.65E_{{\text{s}}} }}{{(1 - \mu^{2} )D}}\sqrt[{12}]{{\frac{{E_{{\text{s}}} D^{4} }}{{(EI)_{{{\text{eq}}}} }}}}$, where *D* is the outer diameter of the tunnel; *μ* is Poisson’s ratio of the soil; *E*_s_ is the compressive modulus of the foundation soil; and (*EI*)_eq_ is the equivalent flexural stiffness of the tunnel^37^.

#### Fourier expansion of horizontal displacement curve function of shield tunnel

The distribution of the excavation area in the simplified model in this paper is parallel to the side shield tunnel. In theory, the longitudinal deformation of the shield tunnel should be symmetrical about the middle point of the excavation of the foundation pit, so it can be obtained by Fourier series expansion according to the cosine function:

25 $$ w(l) = \sum\limits_{n = 0}^{\infty } {a_{n} } \cos \frac{n\pi l}{{ND_{t} }} = T_{n} (l)A^{T} $$

where $T_{n} (l) = (1 \, \cos \frac{\pi l}{{ND_{{\text{t}}} }} \, \cos \frac{2\pi l}{{ND_{{\text{t}}} }} \, ... \, \cos \frac{n\pi l}{{ND_{{\text{t}}} }} \, )$, $A = (a_{{0}} \, a_{{1}} \, a_{{2}} \, ... \, a_{n} )^{T}$, and *n* is the Fourier expansion series.

### Variational governing equations solving

Based on the principle of minimum potential energy, the total potential energy *E*_p_ takes the extreme value of each undetermined coefficient,

26 $$ \frac{{\partial E{\text{p}}}}{{\partial a_{i} }} = \frac{{\partial W_{{\text{L}}} }}{{\partial a_{i} }} + \frac{{\partial W_{{\text{R}}} }}{{\partial a_{i} }} + \frac{{\partial W_{{\text{S}}} }}{{\partial a_{i} }} + \frac{{\partial W_{{\text{T}}} }}{{\partial a_{i} }} = 0\quad (i = {0},{1},{2} \ldots n) $$

where *a*_*i*_ is the (*i* + 1)th element in matrix A, i.e., the tunnel deformation curve function coefficient polynomial.

The governing equation can be obtained by solving the above equation:

27 $$ \mathop {}\nolimits_{{}}^{{}} \int_{{ - ND_{t} }}^{{ND_{t} }} {P_{{{\text{ax}}}} \left( l \right)} \left\{ {T_{n} \left( l \right)} \right\}^{{\text{T}}} {\text{d}}l = \left\{ {\sum\limits_{m = - N}^{N - 1} {\left[ \begin{gathered} \left( {k_{{{\text{sl}}}} \left( {1 - j_{{\text{x}}} } \right)^{2} + \frac{{k_{{\text{t}}} j_{{\text{x}}}^{2} D^{2} }}{{3D_{{\text{t}}}^{2} }}} \right) \hfill \\ \frac{{\partial \left( {w\left( {(m + 1)D_{{\text{t}}} } \right) - w\left( {mD_{{\text{t}}} } \right)} \right)}}{{\partial a_{i} }} \hfill \\ \left( {T_{n} \left( {\left( {m + 1} \right)D_{{\text{t}}} } \right) - T_{n} \left( {mD_{{\text{t}}} } \right)} \right) \hfill \\ \end{gathered} \right]} + \int_{{ - ND_{t} }}^{{ND_{t} }} {kD\frac{\partial w(l)}{{\partial a_{i} }}T_{n} \left( l \right){\text{d}}l} } \right\} \times A^{{\text{T}}} $$

Calculated by Eq. (27), the undetermined coefficient matrix *A*^T^ can be obtained:

28 $$ A^{{\text{T}}} = \left( {\left[ {K_{{\text{r}}} } \right] + \left[ {K_{{\text{s}}} } \right]} \right)^{ - 1} \left[ {P_{{{\text{ax}}}} } \right]^{{\text{T}}} $$

The horizontal displacement distribution function of the tunnel axis next to the foundation pit can be obtained by substituting the undetermined coefficient matrix *A*^T^ back into Eq. (24):

29 $$ w(l) = T_{n} (l)A^{{\text{T}}} $$

Equations (17)–(29) are cited from Zhang et al.^15^

## Analysis of Horizontal Displacement of Shield Tunnel Outside Foundation Pit

### Case 1

A foundation pit is located next to the shield tunnel of Hangzhou Metro Line 2, where *L* = 68 m, *B* = 72 m, excavation depth *d* = 15.8 m, and the underground diaphragm wall is 37.2 m below the ground. The minimum distance between the sideline of the foundation pit envelope and the tunnel axis is *s* = 12.6 m^31^. The outer diameter of the shield tunnel lining is *D* = 6.2 m. Using C50 concrete segments, thickness *t* = 0.35 m, and ring width *D*_t_ = 1.2 m. The segment rings are connected by sixteen M30 longitudinal bolts. According to the calculation, *k*_sl_ = 2.23 × 10^6^ kN/m, *k*_t_ = 9.39 × 10^5^ kN/m, and (*EI*)_eq_ = 1.1 × 10^8^ kN m^2^. The tunnel axis is buried at a depth of 14.3 m. The soil in the project site is composed of silty clay, sandy silt, silty sand mixed with silt, and muddy silty clay from top to bottom. The compression modulus of each soil layer is 5.2 MPa, 10 MPa, 12.6 MPa and 3 MPa. The soil layers within the excavation area of the foundation pit from top to bottom are silty clay, sandy silt and silty sand mixed with silt, respectively. The tunnel is predominantly passing through the sandy silt, silty sand mixed with silt and muddy silty clay. According to the actual engineering geological conditions, Poisson's ratio of the soil is *μ* = 0.4 and *E*_s_ = 6.39 MPa. The measured data of tunnel deformation is from the literature^31^.

**Figure 11:**
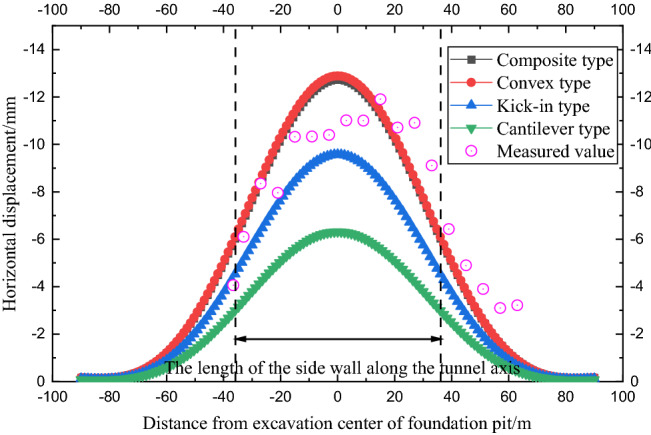
Comparison of tunnel deformation caused by different deformation modes of retaining structure (case 1).

In the calculation example, *δ*_max_/*d* = 0.6%, *N* = 300, and the proportional coefficient of the rotation effect of the shield tunnel *j*_x_ = 0.2. Figure 11 shows the comparison curve between the horizontal displacement of the tunnel calculated by the four envelope deformation modes and the measured value.

As shown in Fig. 11, when the composite and convex deformation modes of the envelope structure occur, the horizontal displacement curves generated by the tunnel are almost the same and are in good agreement with the measured horizontal displacement of the tunnel, which verifies the correctness of the calculation method in this paper. The kick-in deformation mode and cantilever deformation mode underestimate the horizontal displacement of the tunnel. The influence range of tunnel deformation caused by the four envelope deformation modes is similar at approximately twice the length of the sidewall of the foundation pit along the tunnel axis.

**Figure 12:**
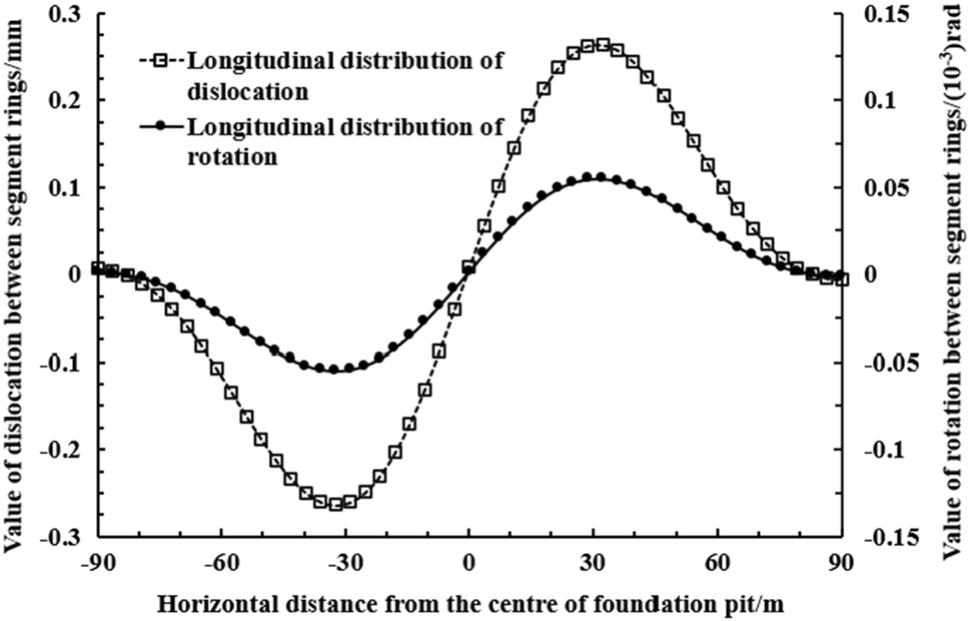
Longitudinal distribution of the amount of dislocation between the rings and the corners between the shield tunnels (convex type).

As shown in Fig. 12, when the convex deformation mode of the envelope structure occurs, segment displacement and inter-ring rotation angle at the maximum horizontal displacement of the side tunnel corresponding to the excavation center of the foundation pit is close to 0. Almost no misalignment deformation and relative rotation deformation. The maximum dislocation between segment rings is 0.26 mm, and the maximum rotation is 5.5 × 10^−5^ rad.

**Figure 13:**
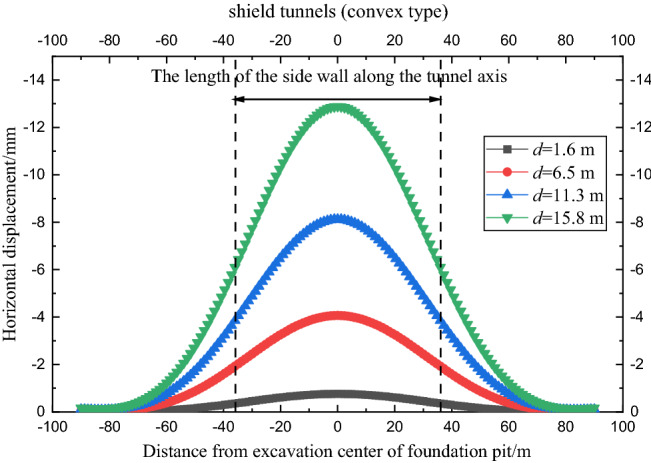
Horizontal displacement curve of shield tunnel under different excavation depths (convex type).

**Figure 14:**
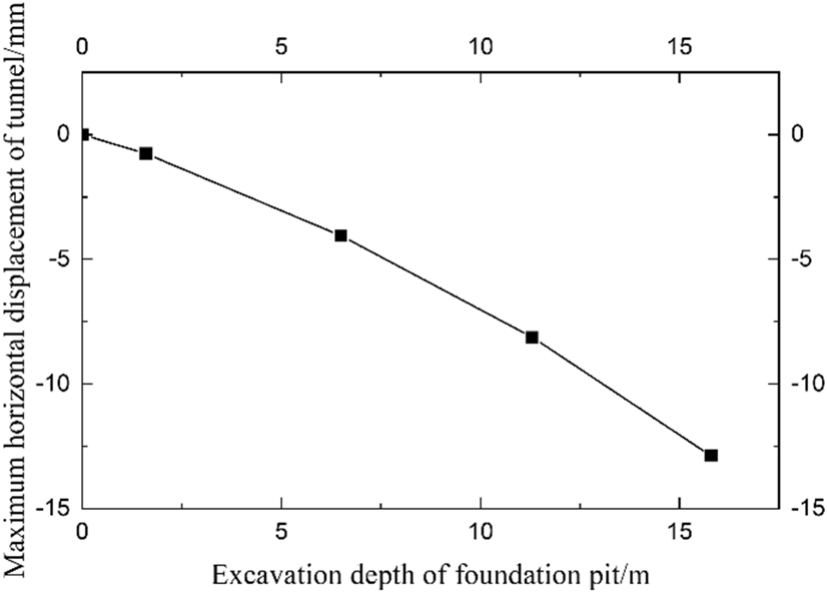
Maximum horizontal displacement of the tunnel under different excavation depths (convex type).

Figure 13 shows the horizontal displacement curve of the tunnel under different excavation depths of the foundation pit. The increase in excavation depth of the foundation pit leads to the deformation of the enclosure structure increase, therefore, the horizontal displacement of the tunnel gradually increases. As shown in Fig. 14, the rate of increase of the maximum horizontal displacement of the tunnel will become larger when the excavation depth of the foundation pit is close to the burial depth of the tunnel.

### Case 2

The plan size of the new office building pit of Hengfeng Bank is 85 m × 45 m, excavation depth is 10.6 m^38^. Suzhou Line 1 is located on the south side of the pit, parallel to the pit, with a distance of about 10.5 m from the enclosure structure, and the tunnel axis burial depth is about 13 m; calculation parameters *k*_sl_ = 2.23 × 10^6^ kN/m, and *k*_t_ = 9.39 × 10^5^ kN/m. Within the excavation area of the foundation pit, there are mainly powdered clay mixed with clay and silty. The tunnel interval is located in powdered clay with soil Poisson's ratio *μ* = 0.35 and *E*_s_ = 10.76 MPa. The measured data of tunnel deformation is from the literature^38^.

In the calculation example, *δ*_max_/*d* = 0.5%, *N* = 100, and the rotational effect proportional coefficient of the shield tunnel beside the foundation pit is *j*_x_ = 0.2. Figure 12 shows the comparison curve between the horizontal displacement of the tunnel calculated by the four envelope deformation modes and the measured value.

**Figure 15:**
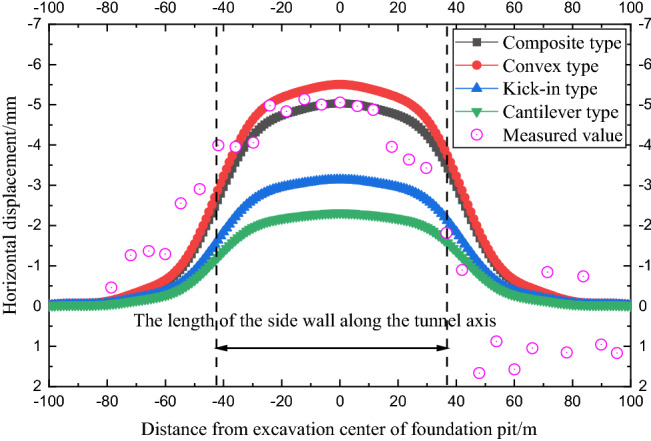
Comparison of tunnel deformation caused by different deformation modes of retaining structure (case 2).

As shown in Fig. 15, under the working conditions of case 2, the horizontal displacement law of the side shield tunnel caused by the four deformation modes of the foundation pit envelope is similar to that of case 1.

The horizontal displacement distributions of the shield tunnel caused by the convex type and the composite type are similar, and the horizontal displacement of the tunnel under the two deformation modes is in good agreement with the measured value. Similarly, the kick-in and cantilever deformation modes underestimate the horizontal displacement of the tunnel to a certain extent. In case 2, the influence range of the tunnel deformation caused by the four envelope structure deformation modes is also approximately twice the length of the sidewall of the foundation pit along the tunnel axis.

With the development of pit design technology, the support scheme of the adjacent existing shield tunnel gradually develops into a strong support scheme with a large stiffness enclosure structure and multiple internal supports, and the deformation mode of the enclosure structure is mainly composite as well as convex type. In this paper, the construction time of case 1 is 2014 and the construction time of case 2 is 2017, so composite, as well as convex calculation results in the two cases, are more consistent with the actual measurement results.

## Analysis of maximum horizontal displacement of shield tunnel outside foundation pit

**Figure 16:**
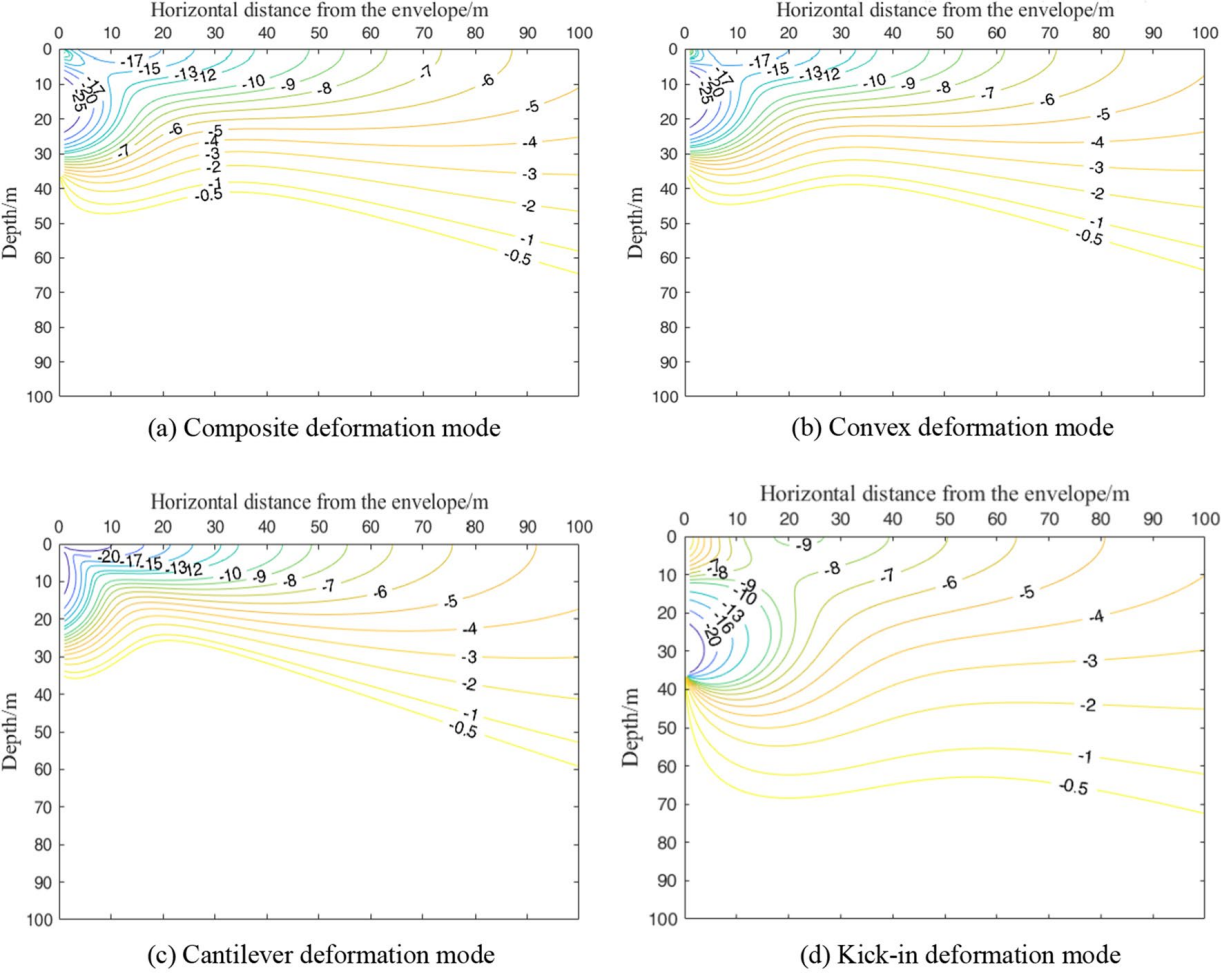
Contour map of maximum horizontal displacement of the tunnel under different deformation modes.

Taking engineering case 1 as the basic working condition, Fig. 16a and b show the maximum horizontal displacement contour of the shield tunnel outside the foundation pit under the deformation mode of the composite and convex enclosure structures, respectively. The distribution pattern of the isogram of the maximum displacement of the tunnel outside the pit caused by the composite as well as the convex deformation mode is similar. The technical specification of urban rail transit engineering monitoring specifies the control value of horizontal displacement of existing tunnels as 3–5 mm^39^. In this section, the horizontal displacement control value of the tunnel is 10 mm. In the composite envelope deformation mode, the tunnel horizontal displacement of more than 10 mm is approximately 46 m from the horizontal distance of the envelope structure, the depth is 30 m within the triangular area, and the range is similar to the convex envelope deformation mode. Both deformation modes reach the maximum horizontal displacement of the tunnel in the area near the pit bottom. In the composite and internal convex deformation modes, the maximum horizontal displacement of the tunnel decreases with increasing horizontal distance and depth from the enclosure, and the effect of depth is significantly greater than the effect of horizontal distance from the enclosure.

Figure 16c shows the contours of the maximum horizontal displacement of the tunnel outside the pit under the deformation mode of the cantilever-type enclosure structure. As shown in Fig. 16c, the area where the horizontal displacement of the tunnel is greater than 10 mm is approximately 43 m from the horizontal distance of the enclosure structure and the triangular area within 23 m in depth, which is further reduced compared with the area under the composite and internal convex deformation modes. As the deformation of the upper part of the enclosure reaches its maximum in the cantilever-type deformation mode, the soil near the upper area is disturbed more, and the area where the maximum displacement of the tunnel reaches its maximum is near the upper part of the enclosure.

Figure 16d shows the contours of the maximum horizontal displacement of the tunnel outside the pit in the deformation mode of the kick-in type enclosure. In the kick-in type enclosure deformation mode, a tunnel horizontal displacement of more than 10 mm in the area is about to the toe of the enclosure as the center of the circle, the radius of a 16-m semicircle area. Compared with the other three envelope deformation modes, the kicker deformation mode has a smaller range in the horizontal direction and an increased depth in this area. The reason is that the kick-in type deformation mode enclosure reaches the maximum deformation at the bottom, the area with the largest soil deformation is near the bottom of the enclosure, and the influence depth increases compared with the other three deformation modes.

## Conclusion

Considering the deformation mode of the retaining structure, this paper proposes a simplified calculation method of the horizontal displacement of the shield tunnel caused by the excavation of the foundation pit, which can accurately evaluate the influence of the excavation of the foundation pit on the deformation of the tunnel. From the case conditions of this paper, the following four main conclusions are drawn.

1. The horizontal displacement fields of soil caused by the deformation modes of composite and convex enclosures are similar in distribution. Except for the horizontal displacement of the soil outside the pit caused by the cantilever-type deformation mode, which always shows a "cantilever-type" curve, the horizontal displacement of the deep soil outside the pit caused by other deformation modes develops from the "bow" to the "cantilever-type" curve with increasing horizontal distance from the enclosure structure.
2. The vertical displacement field of the soil outside the pit caused by the deformation mode of four enclosure structures shows a "spoon" shape. The horizontal influence range of soil settlement caused by the four modes is, in descending order, kick-in, composite, convex, and cantilever type. After reaching critical depth, the soil will show a certain uplift deformation. The critical depths of the composite type and convex type are similar, while the kick-in type is the largest and the cantilever type is the smallest.
3. For the four deformation modes on the horizontal displacement impact area of the tunnel outside the pit, the impact area of the convex type and the composite type is basically the same. However, since there is a certain displacement at the top of the composite enclosure, the range in the horizontal direction is slightly larger than that of the convex type. The cantilever mode has the smallest influence area, while the kick-in mode has the largest deformation at the bottom of the envelope, and the influence area is smaller in the horizontal direction but increases in the depth direction compared with the other three deformation modes.
4. There is a large difference in the deformation of the side shield tunnel caused by different envelope deformation modes for the same cumulative maximum deformation of the envelope structure. During the construction of the foundation pit, the maximum displacement of the enclosure structure does not completely reflect the impact of the enclosure structure deformation on the tunnel outside the pit. In the predesign stage of the pit, it should be combined with the relative position of the pit and the tunnel to design a suitable support scheme, control the deformation mode of the enclosure structure and minimize the impact on the surrounding environment.

## Discussion

IN this research, the influence of different deformation modes of foundation pit retaining structures on the deformation of the surrounding soil is analyzed. At the same time, a simplified calculation method for the deformation of the side shield tunnel caused by the excavation of the foundation pit is proposed, which can calculate the rotation and dislocation of the shield tunnel during the deformation process.

Compared with previous studies, the calculation method in this research has the following advantages:

1. The previous research^4–6,11,14,15^ on the calculation method of shield tunnel deformation beside the foundation pit used the stress release amount of the side wall of the foundation pit to evaluate the tunnel deformation. However, the determination of the stress release amount is difficult and cannot be verified. The method in this paper evaluates the deformation of the side tunnel by the deformation of the side wall, which can be determined by the measured engineering data or the specified deformation control value and can be easily verified.
2. As the calculation results in this paper show (Figs. 8, 9, 11, 15, 16), when the maximum deformation of the enclosure structure is the same, the deformation of the surrounding soil and the adjacent shield tunnel is greatly different due to different deformation modes. The calculation method in this paper can consider the deformation mode of enclosure structures, which is more consistent with the actual situation of the engineering.
3. Most previous studies^4–6,11,14^ simplified the shield tunnel to the Euler–Bernoulli beam or Timoshenko beam, but could not consider the characteristics of shield tunnel segment assembly. In this paper, the rotating staggered platform model is introduced to simulate the rotation and staggered platform behavior of the shield tunnel segment during deformation.

However, the calculation method in this paper still has some shortcomings:

1. Many simplifications are made in the analysis process, such as ignoring the influence of tunnel existence, precipitation, stratification of foundation soil and nonlinear action of tunnel and soil. No consideration is given to the nonuniformity of soil convergence, and the theoretical results will have certain errors, which can be further studied based on this study.
2. The parameter *δ*_max_/*d* plays an important role in the calculation results. There are three methods to take the value of parameter *δ*_max_/*d*, namely, the measured data, the control value specified in the specification or design, and the parameter back analysis. To accurately evaluate the deformation of the side shield tunnel caused by the excavation of the foundation pit, the accurate value of parameter *δ*_max_/*d* is necessary. However, due to the regional differences in the soil mass and the different supporting conditions of the foundation pit, the parameter *δ*_max_/*d* of different projects varies greatly. The subsequent statistical analysis of the parameter *δ*_max_/*d* of different soil mass conditions and different regions will be carried out to help the accurate value.

## Data Availability

The authors declared that “All data generated or analyzed during this study are included in this published article”.
